# Optical Coherence Tomography and ImageJ Software for objective assessment of optical density of anterior chamber iris cyst

**DOI:** 10.22336/rjo.2021.50

**Published:** 2021

**Authors:** Avadhesh Oli, Shrikant Waikar, Robin Malik, Rupali Bhirud

**Affiliations:** * Department of Ophthalmology, Institute of Naval Medicine, Indian Naval Hospital Ship Asvini, Colaba, Mumbai, India

**Keywords:** iris cyst, optical density, ImageJ for iris cyst, Optical Coherence Tomography

## Abstract

**Aim:** To report the optical characteristics of the fluid in an anterior chamber iris cyst.

**Method:** A 26-year-old male presented with blurring of vision in his right eye for two months, without any other associated ocular complaints. His visual acuity was 6/ 9 on Snellen’s chart. On slit lamp examination, a small translucent pigmented cyst was noted inferiorly in the anterior chamber, struck on to the cornea at 6 o’clock periphery, without any feeder vessel. Anterior segment optical coherence tomography (OCT) revealed a cystic lesion with hyperreflective walls and hypo reflective lumen attached to the cornea, compressing the endothelium.

**Results:** The optical density (OD) of the cyst fluid was determined using ImageJ, an open code Java-based image processing software. The OD of cyst fluid was found comparable to the anterior chamber fluid.

**Conclusion:** Ultrasound biomicroscopy (UBM) and OCT are useful tools for the diagnosis of cystic lesions of the anterior segment. The innovative use of an OCT image and the ImageJ software to determine the optical density of the iris cyst may aid in the diagnosis and follow-up of such cases.

## Background

Iris cysts have always fascinated ophthalmologists because of rarity of occurrence and mystery about their origin. The aetiology of primary iris cysts is poorly understood, whereas secondary cysts have an antecedent cause like the use of miotics, trauma or surgery [**1**]. Primary iris cysts are clinically classified as epithelial and stromal subtypes. Epithelial cysts arise from layers of pigmented epithelium of the iris. Additionally, based on the location, cysts can be central, middle, or peripheral in relation to the pupil. The iris pigmented cysts can present at the pupillary margin, posterior layers of iris epithelium or free-floating in aqueous or vitreous [**2**]. 

Iris cysts in the anterior chamber have been well described in literature. These cysts are lined with pigment epithelium and contain clear fluid. They can be free-floating in the anterior chamber or fixed to anatomical structures. Usually, these cysts remain asymptomatic, but can lead to complications due to inflammation, glaucoma, or mass effect. At times, these mass lesions have been mistaken for malignancy, and literature has few reports of enucleation performed for the fear of malignancy [**3**]. The clinical and morphological features of such cysts are well characterized; however, the optical properties of the contents are not well documented. ImageJ software has been used in retinal images to characterize the optical density of subretinal fluid in various retinal diseases [**4**]. This report describes for the first time the optical density (OD) of an anterior chamber iris cyst using optical coherence tomography (OCT) and ImageJ software. 

## Case presentation

A 26-year-old male presented with complaints of blurring of vision in his right eye (RE) for past two months without any other ocular complaints. He denied any history of trauma or surgery. Institutional ethical clearance was taken for the study and it adhered to the 1975 Helsinki Declaration, as revised in 2000 and 2008. Consent was taken from the patient for examination and use of images, and health data for the purpose of research and publication.

**Fig. 1:**
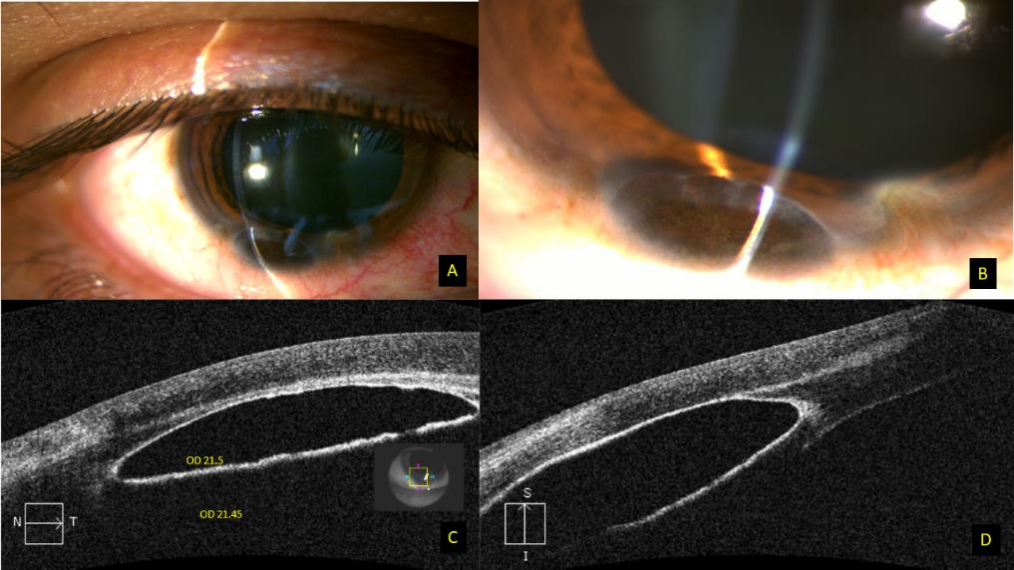
**A, B** Slit-lamp photograph of the right eye showing a pigmented translucent cyst in the anterior chamber, struck to the cornea at 6 o’clock; **C, D** OCT scan showing a cystic lesion in the anterior chamber with hyperreflective walls and clear hypo reflective lumen attached to the cornea. The optical density on ImageJ software is comparable to anterior chamber fluid

His visual acuity was 6/ 9 in RE improving to 6/ 6 with 0.5 Dcyl at 800 and 6/ 6 in the left eye. On slit-lamp examination, anterior segment in the RE showed a small pigmented, cystic, translucent lesion located inferiorly in the anterior chamber, struck to the corneal endothelium at 6 o’clock, without any feeder vessel. The cyst measured 4 mm horizontally, 2 mm vertically and 1.5 mm antero-posteriorly, with the anterior border of cyst abutting corneal endothelium and posterior border extending up to the iris stroma (**Fig. 1 A, B**). The rest of the anterior and posterior segment examination in the right eye was within normal limits, and left eye examination was unremarkable. The intraocular pressure was 18 and 16 mmHg by non-contact tonometry in right and left eye respectively. On further inquiry, the patient admitted that he has noticed the colored spot since childhood and that it has been static since then, with no ocular complaints.

Anterior segment OCT revealed a cystic lesion in the anterior chamber, with hyperreflective walls and clear hypo-reflective lumen attached to the cornea compressing the endothelium (**Fig. 1 C, D**). Gonioscopy showed open angles in all four quadrants in both eyes. The optical density (OD) of the fluid was determined using ImageJ, an open code Java-based image processing software available at http://rsb.info.nih.gov/ij/index.html), using the procedure described earlier [**5**]. The optical density of the cyst was 21.5, which was comparable to anterior chamber fluid, suggesting clear contents inside the cyst (**Fig. 1 C, D**).

As the patient was asymptomatic, he was advised to present to follow-up every year to monitor the progression of the cyst. He was prescribed spectacles for the correction of his astigmatism in the RE and was counselled about the natural history of the cyst.

## Discussion

Pigment epithelial cysts of the iris may be seen in the anterior chamber or in the vitreous cavity as free-floating cysts. They have a brown/ black pigmented, velvety appearance and typically do not transilluminate. In this rather unusual case of primary congenital cyst of the iris, it appears that the cyst got dislodged into the anterior chamber and settled inferiorly due to gravity. The OCT and ImageJ software could help in establishing the optical properties of the contents. 

Primary stromal cysts arise from ectopic surface epithelium, trapped in the iris during embryological development, while secondary iris cysts arise after ocular trauma or surgery [**2**]. While evaluating an iris cyst, it is crucial to rule out iris melanoma, which appears as a brown or variably colored lesion arising from the stroma, maintaining constant contour even after pupillary dilation and possibly having sentinel or intrinsic vessels [**6**]. Ultrasound biomicroscopy (UBM) is usually the recommended modality for the imaging of iris cysts, which displays high reflectivity of the cyst wall; however, OCT characteristics are not well described in literature [**7**]. As documented in this report, the OCT findings substantiate similar findings, as noted in the UBM studies. The outer wall of the cyst is hyperreflective, with clear hypo reflective lumen. Besides, OCT has advantages over UBM, as it is a rapid, non-contact, and non-operator dependent imaging modality to document the characteristics of the cyst and the surrounding ocular tissues.

In this report, we adopted an innovative approach to characterize the optical properties of the fluid inside the cyst using ImageJ software [**5**]. The optical density describes the amount of light received by the sensor while passing through a particular medium. We found that the optical density of the cyst fluid was comparable to the anterior chamber fluid. This innovative method could be used for objective assessment and follow-up of such cases, as any change in the contents would be picked up objectively at the initial stage by the change in OCT characteristics and optical density. 

The cysts at the margin of the pupil are called floccule and are believed to be caused due to a genetic defect in smooth muscles [**6**]. Such patients should be advised to undergo a cardiological evaluation, as the same defect in smooth muscles may lead to aortic dissection. 

Treatment of iris cysts depends on the location and symptoms of the individual. The long-term complications of iris cyst include obscuration of the visual axis, corneal decompensation, secondary uveitis, and glaucoma. Treatment options range from observation to fine-needle aspiration, laser (argon, Nd:YAG), or surgical excision. However, a step ladder approach should be adapted, and treatment should be individualized based on the clinical picture. The OCT seems to be a promising tool for imaging the cyst and optical properties of the fluid on imageJ software may prove to be an objective method, useful for the diagnosis and follow-up of such patients, as it was recently reported in capsular block distension syndrome [**7**].

## Conclusion

Ultrasound biomicroscopy and OCT are useful tools for the diagnosis of cystic lesions of the anterior segment. Innovative use of ImageJ software on OCT image to determine the optical density of the cystic lesion may further characterize the iris cyst, as described in this report. The measurement of the optical density of the contents of the cyst may aid in the initial diagnosis and follow-up of such cases, however further investigations are warranted in larger cohorts of patients.

**Conflict of Interest statement**

The authors declare no conflict of interest. 

**Informed Consent and Human and Animal Rights statement**

Informed consent has been obtained from all individuals included in this study.

**Authorization for the use of human subjects**

Ethical approval: The research related to human use complies with all the relevant national regulations, institutional policies, is in accordance with the tenets of the Helsinki Declaration, and has been approved by the review board of Institute of Naval Medicine, Indian Naval Hospital Ship Asvini, Colaba, Mumbai, India.

**Acknowledgements**

None.

**Sources of Funding**

Authors have no competing interests to declare. No financial disclosures. This research did not receive any specific grant from funding agencies in the public, commercial, or not-for-profit sectors. Consent was taken from patient for use of images, health data and OCT scans for the purpose of research and publication. No external funding was received.

**Disclosures**

None.
